# Supplementary material for: Cytokine CCL9 Mediates Oncogenic KRAS-Induced Pancreatic Acinar-to-Ductal Metaplasia by Promoting Reactive Oxygen Species and Metalloproteinases
Source: Int J Mol Sci. 2024 Apr 26;25(9):4726. doi: 10.3390/ijms25094726 (PMC11083758; doi:10.3390/ijms25094726)
Supplement: Supplementary file 1 [file ijms-25-04726-s001.zip › ijms-2919318-supplementary.pdf]

Figure S1

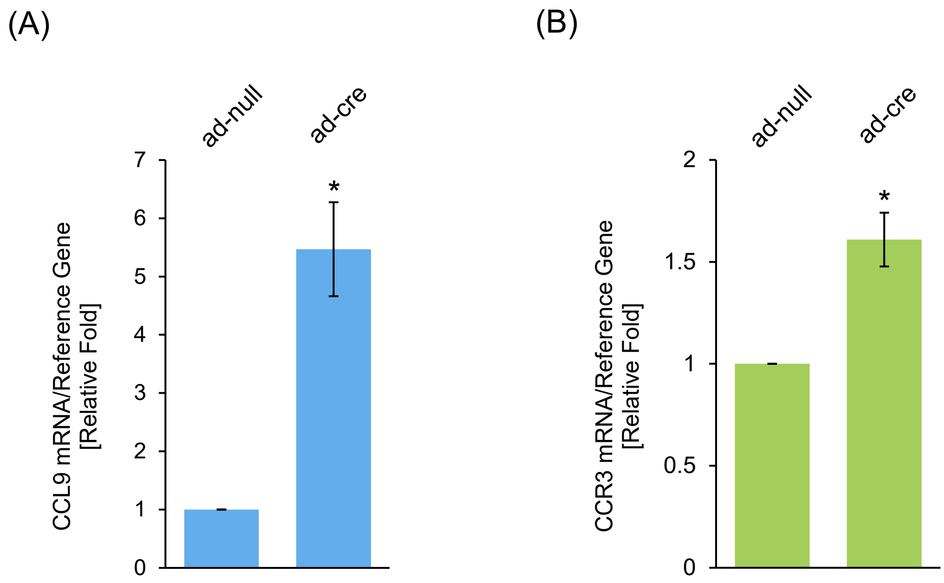

Supplemental Figure S1. Increased levels of mRNA of CCL9 and its receptor CCR3 in 3D ogranoid culture of pancreatic ADM. Primary pancreatic acini of LSL-Kras^{G12D} mouse were infected with either ad-null or ad-cre adenovirus followed by embedded in collagen in 3D. At day 5, the cells isolated from collagen and were collected for real time qRT-PCR for levels of (A) CCL9 mRNA and (B) CCR3 mRNA while a reference gene GAPDH or 18srRNA was used as an internal control. *: p<0.05 as compared to ad-null group.

Figure S2

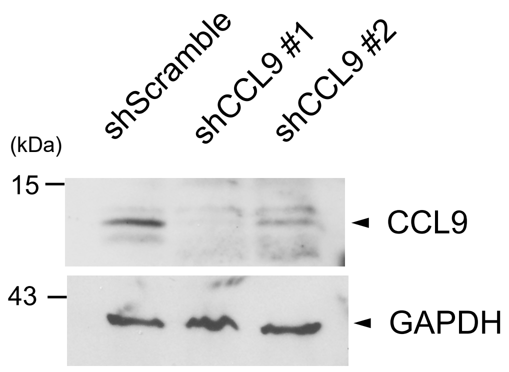

Supplemental Figure S2. Knockdown of CCL9 via shCCL9 lentivirus delivery. Cells were infected with either shScramble (ctrl), shCCL9 #1 or shCCL9 #2 lentivirus before embedded in 3D collagen. The protein levels of CCL9 were evaluated by immunoblots using GAPDH as an internal control.

Figure S3

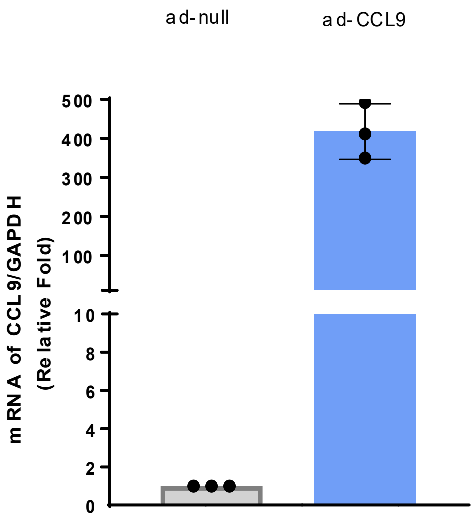

Supplemental Figure S3. Expression of CCL9 in the pancreatic acini via ad-CCL9 adenovirus delivery. Primary pancreatic acini isolated from mouse pancreas were infected with either ad-null or ad-CCL9 adenovirus. The expression of CCL9 at 48 h post-infection was evaluated by real time qRT-PCR using GAPDH as an internal control.

Figure S4

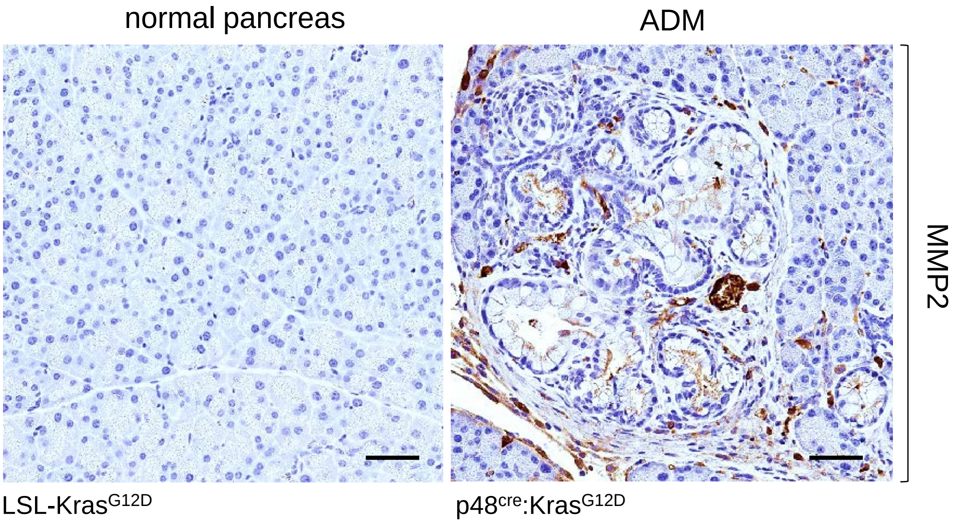

Supplemental Figure S4. MMP2 expression in ADM regions of p48^{cre}:Kras^{G12D} mouse pancreas. The pancreas tissues of p48^{cre}:Kras^{G12D} or LSL-Kras^{G12D} (control) mice were assessed for MMP2 protein expression using immunohistochemistry. scale bar: 50 µm.

Figure S5

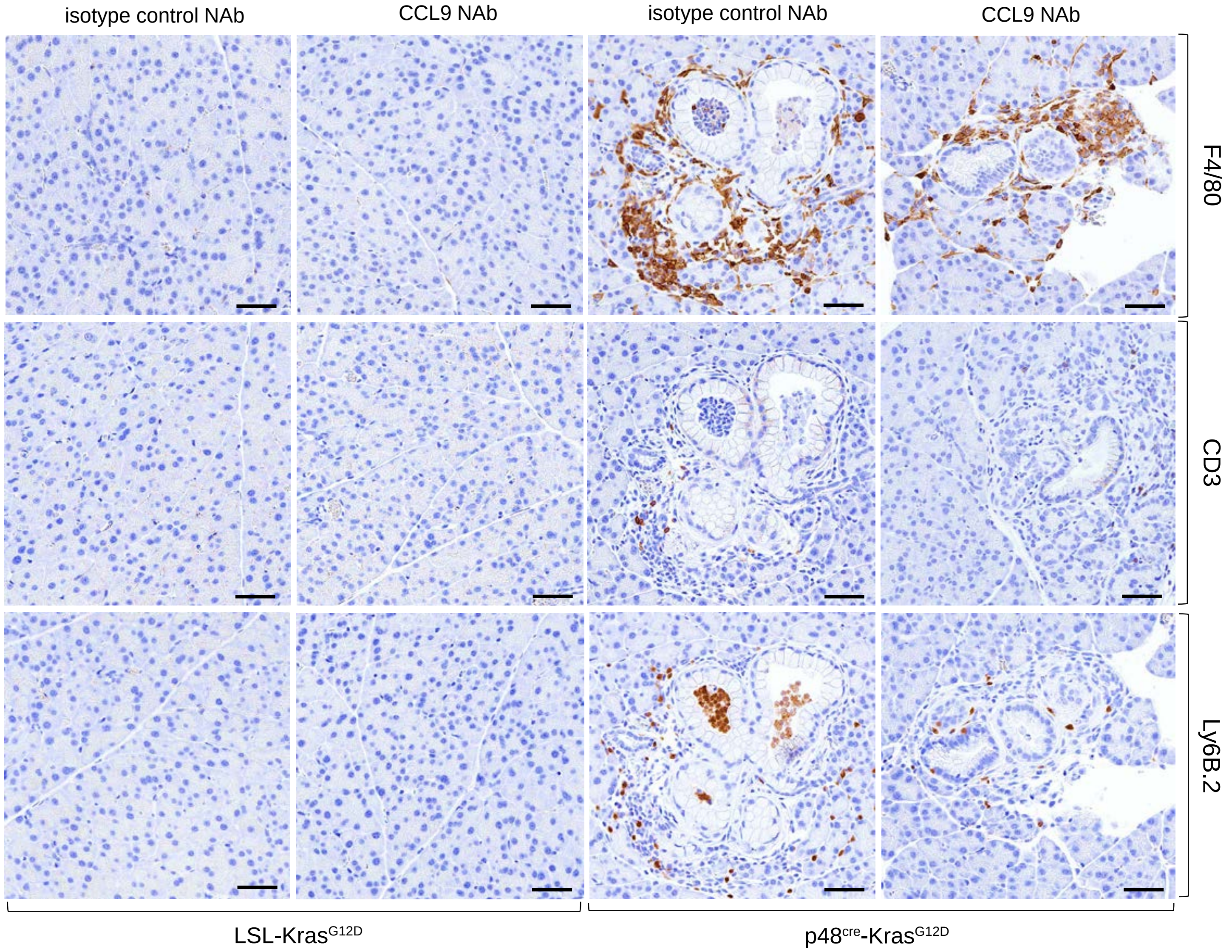

Supplemental Figure S5. Diminished infiltrating immune cells in the pancreatic ADM regions of p48^{cre}:Kras^{G12D} mice treated with CCL9 neutralizing antibody (NAb). Mice of LSL-Kras^{G12D} and p48^{cre}:Kras^{G12D} were treated with either isotype control NAb or CCL9 NAb as described in Materials and Methods. The inflitrating immunce cells including macrophages (F4/80), T cells (CD3) and neutrophils (Ly6B.2) in the pancreatic ADM areas were evaluated using immunohistochemistry. Scale bar: 50 µm.

Figure S6

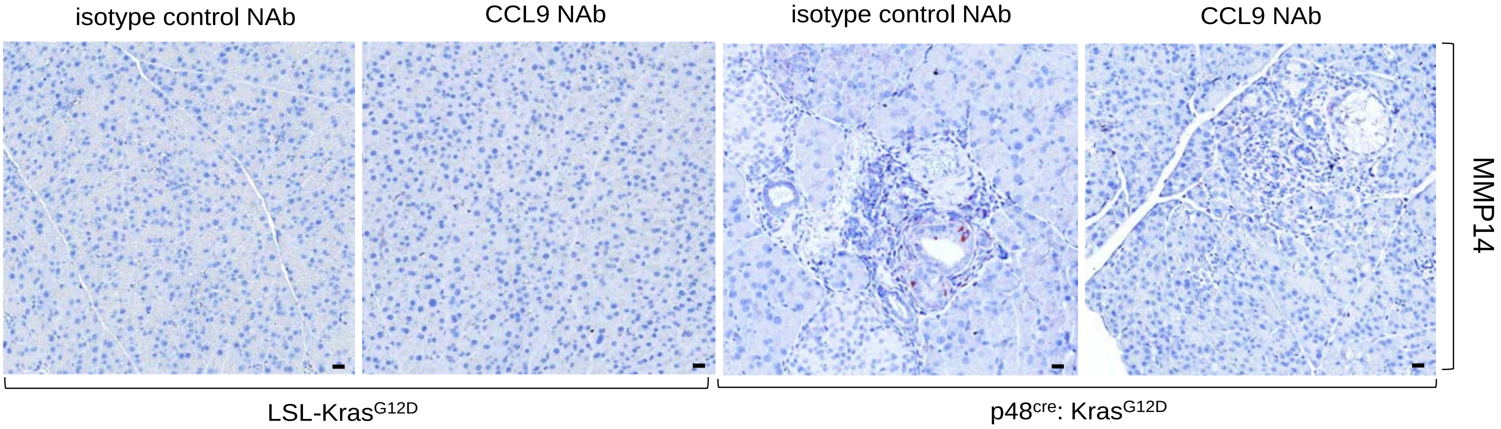

Supplemental Figure S6. Reduction of MMP14 expression in the pancreatic ADM areas of p48^{cre}: Kras^{G12D} mice treated with CCL9 neutralizing antibody (NAb). Mice of LSL-Kras^{G12D} (control) and p48^{cre}:Kras^{G12D} were treated with either isotype control NAb or CCL9 NAb as described in Materials and Methods. The pancreas tissue samples were evaluated for MMP14 expression using immunohistochemistry. Scale bar: 50 µm.

Figure S7

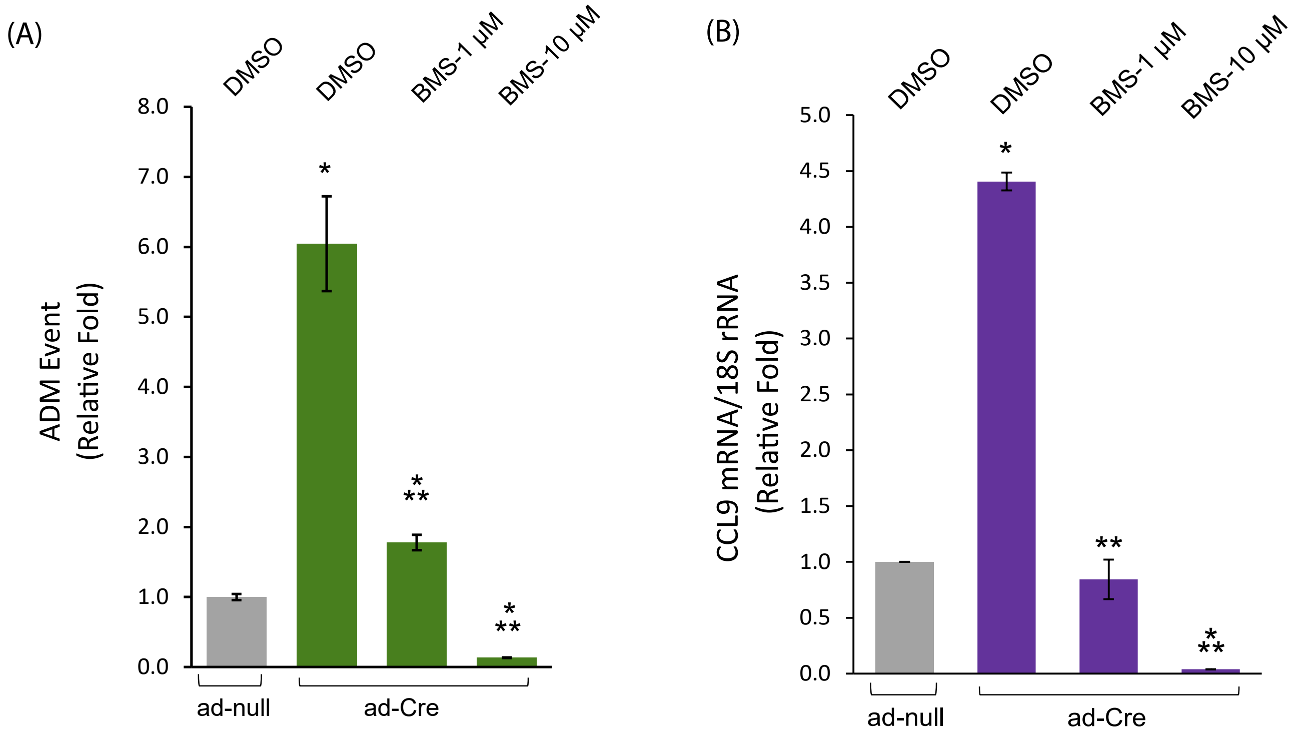

Supplemental Figure S7. Effect of inhibition of NF-κB on Kras^{G12D}-induced ADM and CCL9 trasncripts. Primary pancreatic acini isolated from LSL-Kras^{G12D} mouse were infected with ad-null or ad-Cre in the presence of absence of NF-κB inhibitor BMS345541 with the indicated dose. Acini were then subjected to ADM assay. At the endpoint, ADM events were quantfied and shown in (A). In addition, RNA was isolated at the endpoint and evaluated for CCL9 mRNA levels via real time qRT-PCR (B). *: p<0.05 as compared to ad-null/ctrl+DMSO; **: p<0.05 as compared to ad-Cre+DMSO.
